# Effects of Green Tea Catechins and Theanine on Preventing Influenza Infection among Healthcare Workers: A Randomized Controlled Trial

**DOI:** 10.1186/1472-6882-11-15

**Published:** 2011-02-21

**Authors:** Keiji Matsumoto, Hiroshi Yamada, Norikata Takuma, Hitoshi Niino, Yuko M Sagesaka

**Affiliations:** 1 Department of Drug Evaluation & Informatics, Graduate School of Pharmaceutical Sciences, University of Shizuoka, 52-1 Yada, Suruga-ku, Shizuoka 422-8526, Japan; 2 White Cross Nursing Home, Higashimurayama, Japan; 3 Central Research Institute, ITO EN, Ltd., Shizuoka, Japan

## Abstract

**Background:**

Experimental studies have revealed that green tea catechins and theanine prevent influenza infection, while the clinical evidence has been inconclusive. This study was conducted to determine whether taking green tea catechins and theanine can clinically prevent influenza infection.

**Methods:**

**Design, Setting, and Participants**: A randomized, double-blind, placebo-controlled trial of 200 healthcare workers conducted for 5 months from November 9, 2009 to April 8, 2010 in three healthcare facilities for the elderly in Higashimurayama, Japan.

**Interventions**: The catechin/theanine group received capsules including green tea catechins (378 mg/day) and theanine (210 mg/day). The control group received placebo.

**Main Outcome Measures**: The primary outcome was the incidence of clinically defined influenza infection. Secondary outcomes were (1) laboratory-confirmed influenza with viral antigen measured by immunochromatographic assay and (2) the time for which the patient was free from clinically defined influenza infection, i.e., the period between the start of intervention and the first diagnosis of influenza infection, based on clinically defined influenza infection.

**Results:**

Eligible healthcare workers (n = 197) were enrolled and randomly assigned to an intervention; 98 were allocated to receive catechin/theanine capsules and 99 to placebo. The incidence of clinically defined influenza infection was significantly lower in the catechin/theanine group (4 participants; 4.1%) compared with the placebo group (13 participants; 13.1%) (adjusted OR, 0.25; 95% CI, 0.07 to 0.76, *P *= 0.022). The incidence of laboratory-confirmed influenza infection was also lower in the catechin/theanine group (1 participant; 1.0%) than in the placebo group (5 participants; 5.1%), but this difference was not significant (adjusted OR, 0.17; 95% CI, 0.01 to 1.10; *P *= 0.112). The time for which the patient was free from clinically defined influenza infection was significantly different between the two groups (adjusted HR, 0.27; 95% CI, 0.09 to 0.84; *P *= 0.023).

**Conclusions:**

Among healthcare workers for the elderly, taking green tea catechins and theanine may be effective prophylaxis for influenza infection.

**Trial Registration:**

ClinicalTrials (NCT): NCT01008020

## Background

Influenza infection is the principal cause of acute respiratory illnesses and occurs in epidemics worldwide, in all ages [1]. To reduce morbidity and mortality, a variety of public health interventions have been implemented, including facemasks, gargling, improved hand hygiene and coughing etiquette [2-5]. Vaccines are the most widely used intervention for influenza infection prophylaxis, but their effectiveness depends on the type of influenza virus involved in each season's epidemic, and they also have the drawback of limited supply [6]. Thus far, evidence supporting the effectiveness of antivirals such as amantadine or neuraminidase inhibitors has not been well established [7]. Therefore, it is important to find other ways to reduce the prevalence of influenza infection.

The use of catechins and theanine, which are well-known components of green tea, shows promise as an intervention for preventing influenza infection [8]. Experimental studies have revealed that green tea catechins can prevent influenza infection *in vitro*, but clinical evidence has so far been inconclusive [9]. Recently, in a small prospective cohort study, we reported that gargling with tea catechin extracts was effective in preventing influenza infection in elderly nursing home residents [10]. The consumption of tea extracts including catechins and theanine has also been reported to enhance systemic immunity and prevent the occurrence of upper respiratory tract infection and influenza symptoms in healthy adults [11]. Based on these findings, we designed a randomized, double-blind study to evaluate the clinical efficacy of green tea catechins and theanine in preventing influenza infection.

## Methods

### Study Design

A randomized, double-blind, 2-group parallel study was conducted to compare the effects of green tea catechins and theanine (catechin/theanine) capsules with those of placebo on the prevention of influenza for 5 months during the influenza season, from November 9, 2009 to April 8, 2010. We enrolled adult (over 20 years of age) healthcare workers who worked in 3 healthcare facilities for the elderly in Higashimurayama, Japan. Participants were excluded for the following criteria: tea allergy; history of influenza infection within 6 months before or 24 hours after entering the study; use of any medication or supplement affecting respiratory tract infections; immune disease; severe cardiac, respiratory, renal, or hepatic dysfunction; anemia requiring treatment; pregnancy or lactation.

The participants completed a self-administered questionnaire to assess baseline characteristics including age, sex, body mass index (BMI), smoking and alcohol consumption, and vaccination for the influenza virus. Their tea consumption habits before intervention were also determined, and any tea beverage such as black, green, oolong, or herbal tea intake was restricted to less than 250 mL per day over the entire course of the study.

Eligible participants were randomized by a computer-generated block randomization schema. Participants were provided with coded aluminum bags containing catechin/theanine capsules or placebo identical in appearance and taste in a double-blind manner. The participants were asked to take 6 capsules per day, containing a total of 378 mg catechins (including 270 mg (-)-epigallocatechin gallate) (THEA-FLAN 90S, ITO EN, Ltd., Tokyo, Japan) and 210 mg theanine (Suntheanine^®^, Taiyo Kagaku Co., Ltd., Mie, Japan) or placebo. The participants were asked to complete a questionnaire concerning the occurrence of influenza infection, preventive measures for maintaining hygiene, any adverse event, and their daily adherence to taking the capsules. The questionnaires were collected monthly, and careful safety monitoring was conducted throughout the study. Each participant was observed for the same time.

All participants gave written informed consent before entering the study. The study protocol was approved by the ethics committee at the University of Shizuoka and was conducted in accordance with the Declaration of Helsinki.

### Outcomes

The primary outcome was the incidence of clinically defined influenza infection. The doctor diagnosed clinically defined influenza on the basis of fever (temperature, ≥37.8°C) and any 2 of the following clinical symptoms: cough, sore throat, headache, or myalgia [12]. The secondary outcomes were (1) the incidence of laboratory-confirmed influenza infection with viral antigen measured by immunochromatographic assay (RapidTesta^® ^FLUII, Sekisui Medical Co., Ltd., Tokyo, Japan) and (2) the time for which the patient was free from clinically defined influenza infection, i.e., the period between the start of the intervention and the first diagnosis of influenza infection. The antigen test was performed for all participants with suspected influenza infection, including clinically defined influenza. The antigen test, with a nasopharyngeal swab specimen, was approximately 85% sensitive and 100% specific for the influenza virus type A and B antigens [10,13].

### Statistical Analyses

In consideration of our previous study in the aged-group participants, we estimated that the primary outcome would occur in 1% of participants in the catechin/theanine group and 11% of those in the placebo group [10]. The sample size was calculated as 88 for each group at a power level of 0.80 and a 2-sided α level of 0.05. With an estimated 10% dropout rate, we set the total sample size at 194.

All efficacy and safety analyses were performed according to the intention-to-treat principle. We used Fisher's exact test for categorical comparisons of the data. Differences in the mean values of continuous measurements were tested by Student's *t *test or Wilcoxon rank sum test. Multiple logistic regression analysis was used to provide adjusted odds ratio (OR) estimates and 95% confidence interval (CI) for association between catechin/theanine capsules and the incidence of influenza infection. Cumulative incidence rates were determined by the Kaplan-Meier method. The Cox proportional hazards regression model was used to evaluate the association between catechin/theanine capsules and the time for which the patient was free from clinically defined influenza infection, adjusted for potential confounding variables after confirmation of the proportional hazard assumptions. The cases taken the influenza-free time to be censored were the occurrence of primary outcome and discontinuation. Among baseline characteristics, those with *P *< 0.20 were considered potential confounding variables. They were defined on multiple logistic regression analysis and transferred to the Cox proportional hazard model. In these multivariable analyses, we also added age as a significant explanatory variable.

The threshold for statistical significance was set at *P *< 0.05. Analyses were conducted using R version 2.11.1 (R Foundation for Statistical Computing, Vienna, Austria).

## Results

**Figure 1:**
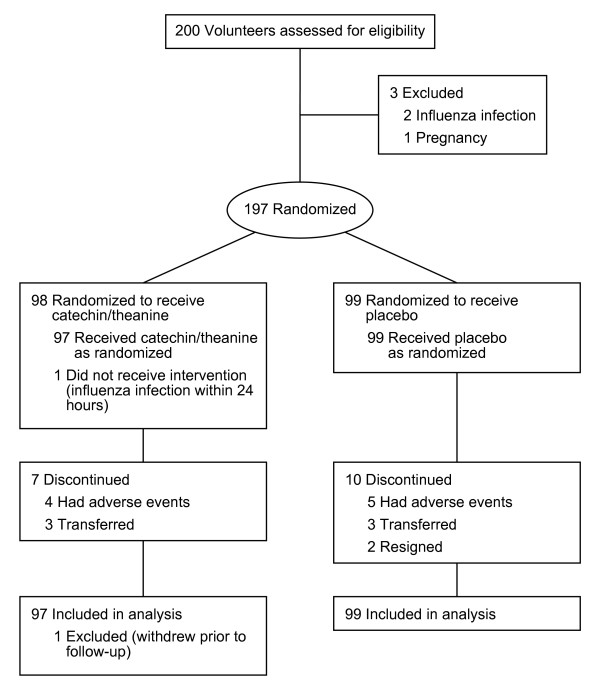
**Flow diagram for trial of catechin/theanine vs. placebo**.

Of 200 participants recruited and assessed for eligibility, 3 were excluded according to the exclusion criteria (2, history of influenza infection within 6 months; 1, pregnancy). The remaining 197 participants were enrolled and randomly assigned to an intervention; 98 were allocated to the catechin/theanine group and 99 to the placebo group (Figure 1). After assignment, 1 participant in the catechin/theanine group was excluded according to the exclusion criteria (influenza infection within 24 hours after entering the study). Adherence to the test capsules was 93.2% in the catechin/theanine group and 91.9% in the placebo group.

The baseline characteristics of the participants are shown in Table 1. The mean age of participants was 42.7 years and uniformly distributed. Study groups were well balanced in terms of baseline characteristics except for BMI (*P *= 0.010). Therefore, BMI was considered as a potential confounding variable and was included in the variables of multivariate analyses. During the study, clinically defined influenza infection occurred in a total of 17 participants (8.7%), 6 of whom (3.1%) were laboratory confirmed with the type A antigen. No participants had more than one influenza infection during the observation time.

**Table 1:** Baseline characteristics of study participants

	Catechin/Theanine group	Placebo group	
Characteristics	(n = 97)	(n = 99)	*P *Value
Age, mean (SD) [range], y	42.1 (12.4) [21-65]	43.2 (13.1) [22-69]	0.63^a^

Sex, No. (%)			
Male	21 (21.6)	23 (23.2)	

Female	76 (78.4)	76 (76.8)	0.86^b^

BMI, mean (SD) [range]	21.6 (2.8) [15.9-30.9]	22.7 (3.2) [16.6-33.3]	0.01^c^

Vaccination for influenza virus, No. (%)	91 (93.8)	91 (91.9)	> 0.99^b^

Hand-washing^d^, No. (%)	72 (74.2)	72 (72.7)	0.87^b^

Facemasks^d^, No. (%)	15 (15.5)	20 (20.2)	0.46^b^

Gargling^d^, No. (%)	71 (73.2)	68 (68.7)	0.53^b^

Smoking, No. (%)			
Yes	29 (29.9)	29 (29.3)	

Past	15 (15.5)	15 (15.2)	> 0.99^b^

No	53 (54.6)	55 (55.6)	

Alcohol consumption, No. (%)			
Yes	49 (50.5)	56 (56.6)	

Past	3 (3.1)	4 (4.0)	0.66^b^

No	45 (46.4)	39 (39.4)	

Tea consumption, mean (SD), mL/day	494.3 (415.6)	481.3 (374.3)	0.95^a^

Abbreviation: BMI, body mass index.

^a ^*P *value based on Wilcoxon rank sum test.

^b ^*P *value based on Fisher's exact test.

^c ^*P *value based on Student's *t *test

^d ^The preventive measures such as hand-washing, facemask application, or gargling was performed 4 days per week or more during the study period.

In the univariate comparison between the incidence of clinically defined influenza infections and baseline characteristics, age was the only significant variable and younger age was correlated with the high incidence of influenza infection (*P *= 0.027). Significant associations were not found among other variables such as sex, vaccination, preventive measures for maintaining hygiene (i.e., hand washing, facemask application, and gargling), smoking, and alcohol and tea consumption.

The incidence of clinically defined influenza infections was significantly lower in the catechin/theanine group (4 participants; 4.1%) than in the placebo group (13 participants; 13.1%) (adjusted OR, 0.25; 95% CI, 0.07 to 0.76, *P *= 0.022). The incidence of laboratory-confirmed influenza infection was also lower in the catechin/theanine group (1 participant; 1.0%) than in the placebo group (5 participants; 5.1%), but this difference was not significant (adjusted OR, 0.17; 95% CI, 0.01 to 1.10; *P *= 0.112).

**Figure 2:**
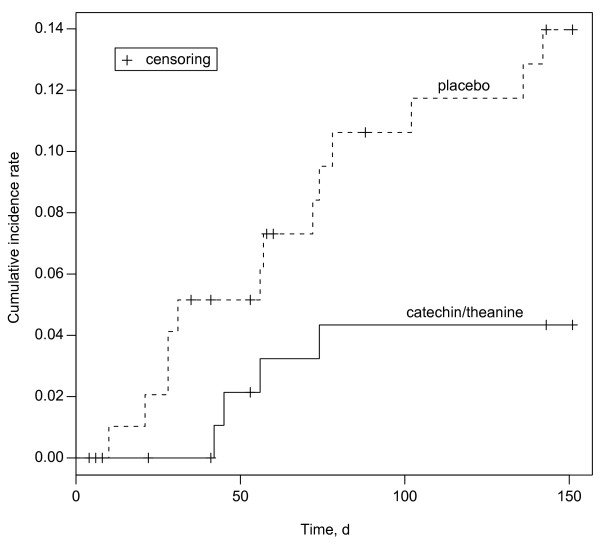
**Kaplan-Meier curves of clinically defined influenza**.

Kaplan-Meier curves were shown in Figure 2. The time for which the patient was free from clinically defined influenza infection, estimated with the Cox proportional hazards regression model, was significantly different between the two groups (adjusted hazard ratio, 0.27; 95% CI, 0.09 to 0.84; *P *= 0.023) (Table 2). No serious adverse events were observed during the study. Digestive symptoms such as bloating and constipation occurred in both groups and were reported by 9.2% of all participants. These symptoms were relativity mild and did not significantly differ between the two groups.

**Table 2:** Results of multiple logistic regression analysis and Cox proportional hazards regression model

	**Laboratory confirmed influenza**^**a**^		**Clinically defined influenza**^**b**^			
Variables	**OR (95% CI)**^**c**^	***P *Value**^**c**^	**OR (95% CI)**^**c**^	***P *Value**^**c**^	**HR (95% CI)**^**d**^	***P *Value**^**d**^
Allocation						
Catechin/Theanine	0.17 (0.01 to 1.10)	0.11	0.25 (0.07 to 0.76)	0.02	0.27 (0.09 to 0.84)	0.02

Placebo	1 [Reference]		1 [Reference]		1 [Reference]	

Age	0.98 (0.91 to 1.04)	0.48	0.95 (0.91 to 0.99)	0.03	0.96 (0.92 to 1.00)	0.04

BMI	0.89 (0.63 to 1.16)	0.43	0.95 (0.79 to 1.11)	0.53	0.97 (0.83 to 1.13)	0.66

Abbreviations: OR, odds ratio; CI, confidence interval; HR, hazard ratio; BMI, body mass index.

^a ^Laboratory-confirmed influenza infection with viral antigen detected by immunochromatographic assay.

^b ^Clinically defined influenza was diagnosed as fever (temperature, ≥37.8°C) and any 2 of the following clinical symptoms: cough, sore throat, headache, or myalgia.

^c ^OR and *P *values were estimated using multiple logistic regression.

^d ^HR and *P *values were estimated using the Cox proportional hazards regression model.

## Discussion and Conclusion

This randomized, double-blind, placebo-controlled trial was conducted to determine whether taking green tea catechins and theanine could clinically prevent influenza infection. We found that consuming catechin/theanine for 5 months had a statistically significant preventive effect on clinically defined influenza infection and was well tolerated. To our knowledge, this is the first randomized clinical trial to evaluate the efficacy of green tea catechins and theanine in the prevention of influenza infection.

Experimental studies have shown some mechanisms of the action of green tea catechins and theanine on the prevention of influenza infection. These studies have shown that green tea catechins bind to the hemagglutinin molecule of influenza virus, thereby inhibiting the virus adsorption to the host cells and blocking virus assembly or maturation cleavage [14-16]. Theanine has a possibility to enhance the systemic immunity (*γδ *T-cell function) for influenza infection [11]. Our results seem to provide clinical evidence to confirm these biological activities.

Contrary to worldwide prevalence in the 2009 novel influenza virus A (H1N1) pandemic, laboratory-confirmed influenza infection occurred in only 3.1% of the study participants. Probably in part due to this small sample size, the incidence of laboratory-confirmed influenza was not significantly different in the catechin/theanine group, in spite of showing a decreased tendency compared to placebo. Moreover, the participants were healthcare workers with a high rate (92.9%) of vaccination; therefore the effectiveness of catechin/theanine might be underestimated. Additional large-scale randomized trials are needed to confirm the effectiveness of catechin/theanine as prophylaxis for laboratory-confirmed influenza infection.

## Abbreviations

OR: odds ratio; CI: confidence interval; HR: hazard ratio.

## Competing interests

This work was supported by a grant from the Japanese Ministry of Health, Labor, and Welfare, and a grant from ITO EN Ltd. ITO EN Ltd played a role in providing the experimental supplements.

## Authors' contributions

KM had complete access to all the data in the study, and he is responsible for the integrity of the data and the accuracy of the data analysis. HY designed the study protocol and participated in its coordination. NT participated in the study coordination. HN and YMS participated in the design of the study and provided the experimental supplements. All authors read and approved the final manuscript.

## Pre-publication history

The pre-publication history for this paper can be accessed here:

http://www.biomedcentral.com/1472-6882/11/15/prepub
